# Towards Greater Standardisation in Benthic Trait Research to Support Application to Environmental Management

**DOI:** 10.1002/ece3.71072

**Published:** 2025-03-04

**Authors:** Irene Susini, Heidi M. Tillin, Louise Anderson, Craig M. Robertson, Sian Rees, Kerry L. Howell

**Affiliations:** ^1^ School of Biological and Marine Sciences University of Plymouth Plymouth UK; ^2^ Plymouth Marine Laboratory Plymouth UK; ^3^ Joint Nature Conservation Committee Peterborough UK; ^4^ School of Ocean Sciences Bangor University Menai Bridge UK

**Keywords:** benthos, biological traits, FAIR principles, standardisation, trait classification, trait‐based approaches

## Abstract

Functional trait‐based approaches have enriched our understanding of key ecological processes such as species assembly and biodiversity loss. This focus on traits, rather than taxonomy, promotes comparability across spatial and organisational scales, further enabling the application of trait‐based methodologies to systems where species identity is difficult to recognise. Among other issues, however, the lack of standardisation is preventing trait‐based approaches from unlocking their true potential. Here, 407 published articles (peer‐reviewed and grey literature) are reviewed alongside the Biological Traits Information Catalogue (BIOTIC) to document inconsistencies in the understanding and use of trait terminology in the context of marine benthic ecosystems. Firstly, discrepancies in the operationalisation of key concepts are noted, each associated with six to ten separate definitions. Secondly, three distinct trait classification frameworks are identified, of which one presents considerable internal variation; within‐framework trait classification also emerges as inconsistent. Lastly, a total of 290 synonyms and associated modalities are noted with respect to 18 traits commonly implemented in benthic research, amounting to an average of 16 synonyms per trait. Researchers should be aware of such inconsistencies; to overcome them, we propose a set of guidelines aimed at standardising the reporting and classification of traits in benthic research for policy and management applications. As other standards may exist, we further present a ‘translation’ table intended for use by trait ecologists when reviewing existing literature that adheres to different trait classification frameworks than the ones we recommend. Standardising the reporting and storage of trait data will help align our understanding of the function of benthic assemblages, their role in delivering ecosystem services, and the impact of human activities on ecosystem function.

## Graphical abstract

Here, 407 published articles (peer‐reviewed and grey literature) are reviewed alongside the Biological Traits Information Catalogue (BIOTIC) to document inconsistencies in the understanding and use of trait terminology in the context of marine benthic ecosystems. Firstly, discrepancies in the operationalisation of key concepts are noted, each associated with six to ten separate definitions. Secondly, three distinct trait classification frameworks are identified, of which one presents considerable internal variation; within‐framework trait classification also emerges as inconsistent. Lastly, a total of 289 synonyms and associated modalities are noted with respect to 18 traits commonly implemented in benthic research, amounting to an average of 16 synonyms per trait.

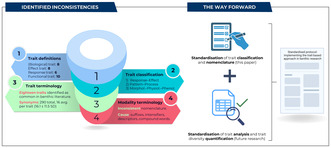

## 1 Introduction

The marine environment is increasingly subject to pervasive anthropogenic pressures, with ca. 59% of global waters experiencing intensifying cumulative impacts (Halpern et al. 2019; Harris 2020). As key components of marine systems, sedimentary benthic communities are regarded as reliable bioindicators of environmental change. They respond to a diverse array of stressors, both anthropogenic and natural, given their occurrence in habitats of highly variable spatiotemporal dynamics (Beauchard 2023). Marine benthos are pivotal for the delivery of key ecosystem functions—that is, fluxes of energy and material in the ecosystem (Bellwood et al. 2019)—on which ecosystem services depend to support human well‐being and livelihoods (Rife 2018). However, the ability of benthos to function effectively is affected directly and indirectly by physical disturbances (e.g., Tiano et al. 2019). For these reasons, benthos have long occupied an integral role in the evaluation framework of European Union (EU) nature legislation to protect habitats (e.g., Marine Strategy Framework Directive, Rice et al. 2012).

Many marine conservation studies to date have focused on the preservation of biodiversity and the protection of vulnerable species (e.g., Rijnsdorp et al. 2018), while disregarding the function(s) that each investigated taxon serves within the broader ecosystem context (Beauchard 2023). It remains uncertain whether the conservation of biodiversity and vulnerable species translates into the preservation of benthic ecosystem functions, since species survival and ecosystem function are not necessarily driven by the same species properties (Díaz and Cabido 2001; Lavorel and Garnier 2002). The loss of biodiversity is always critical, but even more so when the species affected are responsible for important or rare functions within the ecosystem (Beauchard 2023).

To begin to address the biodiversity versus ecosystem function conundrum, it is necessary to first consider the fundamental question of how to understand a species within the context of its ecosystem (Beauchard 2023). Two perspectives relating to the mechanisms underpinning the formation of species assemblages are relevant, namely the Darwinian concept of fitness (growth, survival, and reproduction) and the concept of ecosystem engineering (Jones et al. 1994). Species fitness refers to the phenomenon whereby the biological performances of individual organisms are shaped by the interplay of abiotic forces (i.e., environmental filtering) and biotic interactions (i.e., limiting similarity; Weiher and Keddy 1995). Ecosystem engineering, on the other hand, refers to the phenomenon whereby the expression of the fitness of one species influences the fitness expression of another, for instance through direct modulation of resource availability (autogenic engineering, e.g., habitat creation) or through its indirect counterpart (allogenic engineering, e.g., increase/decrease in oxygen availability through bioirrigation or respiration, respectively). Hence, a taxon can be described with respect to properties that express fitness and/or ecosystem engineering abilities, such properties being collectively referred to herein as biological traits.

The advent of trait‐based approaches has enriched our understanding of key ecological processes such as species assembly, niche differentiation, and ecosystem functioning (e.g., McGill et al. 2006; Cadotte et al. 2015; Schneider et al. 2019). The study of biological traits has proven to be critical in addressing important ecological questions, enabling researchers to add trait information to species identity and explore how the environment directly affects, or is affected by, different organisms based on biological trait information (Weiher and Keddy 1995; Legendre et al. 1997). Such a shift in focus is one of the key premises of trait‐based ecology, promoting comparability across spatial and organisational scales, as well as enabling the application of trait‐based approaches to systems where species identity is difficult to recognise (Messier et al. 2010; Kraft et al. 2015; Carmona et al. 2016). This decoupling from reliance on taxonomy opens the door, at least in principle, to cross‐taxa trait‐based exercises (Shipley et al. 2016). However, the terrestrial vegetation‐centric background of trait‐based ecology has led to most trait theories and approaches being formulated with plants as the model (Violle et al. 2007; Shipley et al. 2016, but see Gravel et al. 2016). This, coupled with the expansion over the past two decades of functional ecology research relying on study organisms that do not conform to the plant life‐form schemes, has engendered inconsistencies in the usage of concepts, definitions, and approaches (Shipley et al. 2016; Brodribb 2017; Dawson et al. 2021). Considering the escalating societal demands placed on benthic ecosystems, there has never been such a pressing need to align our understanding of the relationship between the physical impact of anthropogenic pressures and the function of benthic assemblages. However, it is only by standardising the reporting and storage of trait data that trait‐based approaches will realise their full potential. This paper reviews trait literature in the context of marine benthic ecosystems to understand how biological traits are presented and identify sources of inconsistency. The implications for marine environmental management are discussed, and a ‘translation’ table intended to promote the standardisation of trait classification is proposed.

## 2 Methods

**FIGURE 1:**
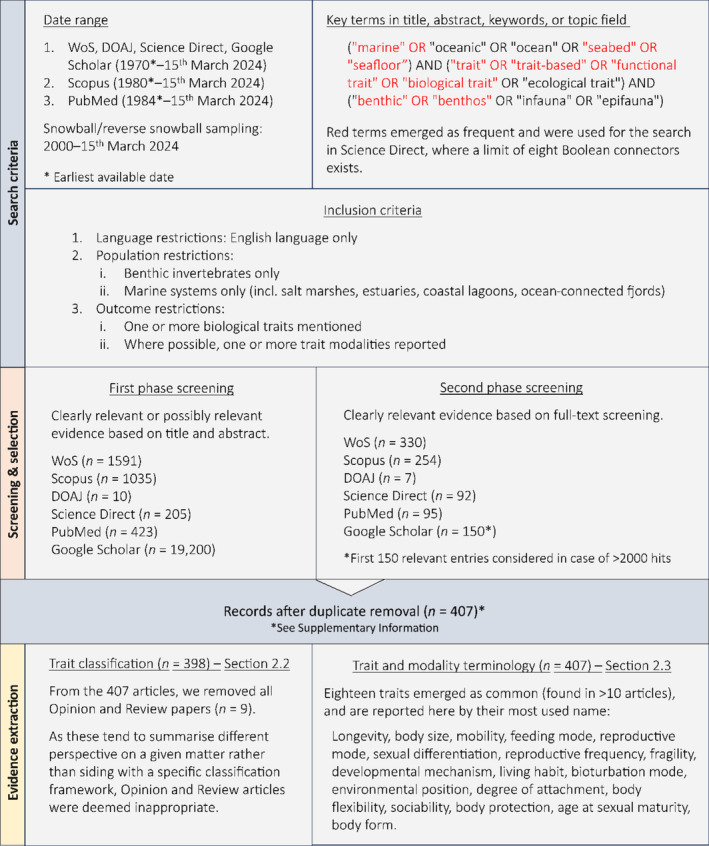
Schematic illustration of the literature search and evidence extraction process.

A hierarchical approach was followed, with investigations focusing first on the identification of all available trait definitions and classification frameworks (if any), progressing with the assessment of trait and modality (i.e., trait values) terminology. In conducting the literature search, the principles of the Preferred Reporting Items for Systematic Reviews and Meta‐Analysis (PRISMA) framework were adhered to (Moher et al. 2010). However, the use of snowball and reverse snowball sampling was necessary to gather evidence on the number of definitions and classification frameworks available in the literature for traits and associated concepts. This was attained through the citation tracking facility offered by large databases that display the articles referenced within a paper of interest as well as the more recent articles citing it. The latter option was particularly useful when dealing with seminal papers, for example, Violle et al. (2007). When using snowball/reverse snowball sampling, the date range was set from the year 2000 to the present day, as formal definitions and classification frameworks began to emerge in the literature during the early 21st century. The rationale behind this deviation from the standard systematic review methodology lies in the fact that the trait‐based approach was originally proposed in the context of plant ecology (Lavorel and Garnier 2002), thus falling outside the remit of the benthic‐focused searches. Moreover, few studies offer definitions or citations in relation to the term ‘trait’ and associated concepts (Dawson et al. 2021), rendering the use of a strict systematic approach impractical. No critical appraisal of the evidence was deemed necessary, as the aim was to obtain a list of existing definitions, classification frameworks, and commonly used benthic traits. With respect to the extracted traits, the decision was made to report them by their most used name so as to ‘accelerate’ terminology standardisation. Three exceptions were made, detailed later in Section 4. A breakdown of the search methodology is presented in Figure 1.

## 3 When the Foundation Stones Are Loose: A Matter of Semantics

### 3.1 What Do You Mean by Trait?

The term ‘trait’ sensu ‘particular feature or distinguishing quality’ has been in use in the English language since the 18th century. Since then, the term has slowly transitioned from its colloquial conception to a more scientific equivalent in a vast array of disciplines. Advancements in community and ecosystem ecology over the past few decades have forced the concept of trait to expand beyond the original notion, leading to the establishment of trait‐based ecology as it is known today. Despite attempts to unify the terminology (e.g., Lavorel et al. 1997; Semenova and van der Maarel 2000; Díaz and Cabido 2001; Violle et al. 2007; Bellwood et al. 2019; Dawson et al. 2021; Streit and Bellwood 2023), there currently remains considerable confusion as to the use of the term ‘trait’ and its underlying concepts. Such uncertainty was not resolved, but rather fuelled, by the introduction of the term ‘functional’ as a modifier of the already ambiguous concept of trait, a move engendered by the emergence of trait‐based ecology—also referred to as functional ecology—as an independent scientific discipline (Calow 1987; Keddy 1992).

Such persistent confusion stems, at least partially, from the plethora of alternate definitions in the literature, examples of which are presented in Table 1. Two well‐known examples are those advanced by McGill et al. (2006) and Violle et al. (2007). McGill et al. (2006) argue that a trait is a distinguishable and quantifiable attribute of an organism that is measured at the individual level and used comparatively across taxa and that a trait becomes functional when it strongly influences organismal performance. Violle et al. (2007), on the other hand, define ‘trait’ as any morphological, physiological, or phenological (M–P–P) property measurable at the individual level and independent of the environment or any other level of organisation. According to the authors, a trait can be regarded as functional when it indirectly influences organismal fitness—sensu Darwin (1859)—via its effect on performance traits, that is, constituents of whole organism performance such as body size (Arnold 1983). Important discrepancies between these two seminal definitions include the reduction in traits to a precise subset of attributes, that is, morphological, physiological, and phenological, by Violle and colleagues, as well as the explicit mention of environmental independence. While some researchers maintain that traits should be assigned at the individual level (McGill et al. 2006; Violle et al. 2007; Lam‐Gordillo et al. 2020a), others argue in favour of measurements occurring at the species level (e.g., Tyler et al. 2012; Shi et al. 2023) or at any other relevant level of organisation (e.g., Volaire et al. 2020; Dawson et al. 2021). The latter has been proposed to enable trait evaluations with respect to life forms lacking defined individuality (e.g., corals, fungi, and mosses) or characterised by individuals acting as a single unit (e.g., insect societies). When considering the conditions that an attribute must fulfil to be classed as a trait, the question of heritability also emerges as the subject of considerable debate (e.g., Dawson et al. 2021). According to Garnier et al. (2016), for example, a trait must be heritable to be regarded as such. However, much like the term ‘trait’, ‘heritability’ has long been part of the English language as a colloquial term to indicate generic causal relationships ascribable to ancestry, in addition to its technical conception in the field of genetics (Visscher et al. 2008). Confusion may thus arise when such strong emphasis is placed on trait heritability, also due to the difficulty inherent to its explicit determination (Dohm 2002; Mitchell 2004). Even from a strictly genetic viewpoint, the heritability qualification may pose significant challenges, as the genetic values upon which heritability depends (sensu broad‐sense, *H* ^2^, and narrow‐sense heritability, *h* ^2^) may vary both spatially and temporally, at times offering estimates of zero heritability. This implies that whether or not a trait is heritable, and, therefore, a true trait, is subject to variability depending on where and/or when the trait is measured, a less‐than‐desirable property (Dawson et al. 2021). Further challenges may arise when dealing with behavioural traits, which are routinely implemented in studies on vertebrate and invertebrate taxa. In this regard, heritability may be defined socially, rather than genetically, or it may even differ depending on scale, for example, assemblage versus population versus multiple populations (Weiss and Ray 2019).

**TABLE 1:** Examples of divergent definitions of the term ‘trait’ and associated concepts.

Term	Definition	Reference(s)	Key inconsistencies
(Biological) trait	Trait: any morphological, physiological, or phenological feature measurable at the species level. Biological trait: a defined and measurable (presence/absence or fuzzy coding) property of an organism, usually at the individual level and used comparatively across species.	Lam‐Gordillo et al. (2020a)	(i) Level of measurement: individual versus species versus other relevant level; (ii) nature of trait: unspecified versus morphological, phenological, physiological, behavioural, cultural versus lifestyle, morphology, behaviour, life cycle versus morphological, physiological, habitat, behavioural, life history.
	Traits are well‐defined, measurable properties of an organism typically assigned at the species level.	Tyler et al. (2012)	
	A trait is a measurable characteristic (morphological, phenological, physiological, behavioural, or cultural) of an individual organism that is measured at either the individual or other relevant level of organisation.	Dawson et al. (2021)	
	A biological trait should reflect lifestyle, morphology, behaviour, and life cycle characteristics.	Paganelli et al. (2012)	
	Traits capture aspects of physiology, morphology, and life history that influence fitness and competitive success.	Litchman et al. (2015)	
	A trait is a variable measured on an organism at any scale, from gene to whole organism, and which can be scaled up from individuals to genotype, population, species, or community.	Volaire et al. (2020)	
	A trait is a well‐defined, measurable characteristic of a species representing its morphology, physiology, phenology, life history, or behaviour, as well as its performance in an ecosystem.	Campanyà‐Llovet et al. (2023)	
	Biological traits are sets of morphological, physiological, habitat, behavioural, and life history features that characterise a species.	Shi et al. (2023)	
Functional trait	A functional trait is one that strongly influences organismal performance.	McGill et al. (2006); Teixidó et al. (2018)	(i) Nature of trait: unspecified versus M‐P–P versus phenotypic versus morphological, behavioural, phenological, structural; (ii) nature of trait‐affected entity: unspecified versus organismal performance versus organismal fitness versus ecosystem properties/processes; (iii) mode of influence on fitness (for relevant definitions): indirect versus direct or indirect; (iv) meaning of ‘functional’: affecting organismal fitness versus linked to the function(s) played by individuals within the community/ecosystem; (v) relationship with the environment: unspecified versus none; (vi) heritability: unspecified versus heritable.
	A functional trait is any M–P–P (morphological, physiological, or phenological) trait which impacts fitness indirectly via its effects on performance traits without reference to the environment or any other level of organisation.	Violle et al. (2007)	
	Physiological, morphological, phenological, and behavioural characteristics which mediate species influences on ecosystem properties and their responses to their environment.	Boyé et al. (2019)	
	A trait arising from or influencing an organism's fecundity, growth, development, or survival, that is, demographic fitness.	Volaire et al. (2020)	
	Functional traits describe a species' influence on ecosystem function (i.e., effect traits) or a species' response to environmental change (i.e., response traits).	Campanyà‐Llovet et al. (2023)	
	Linked to the function(s) an individual plays within a community or ecosystem (used as a synonym of effect trait).	Bremner (2008); Schmitz et al. (2015); Bolam et al. (2016)	
	Aspects of phenotypes at the individual scale that exist along a continuum of response and effect. Functional traits can be physiological, morphological, or behavioural.	Weiss and Ray (2019)	
	Generally understood as heritable properties of the individual that are interrelated through trade‐offs and selected by the environment.	Brun et al. (2017)	
	Any organismal characteristics related to individual performance that can directly or indirectly affect one or more ecosystem properties and processes.	Gusmao et al. (2016)	
	Consisting of all morphological, behavioural, phenological, and structural features that can explain the interaction between species and their environment.	D'Alessandro et al. (2020)	
Response trait	Response traits are phenotypic components that respond to the environment by influencing fitness and organismal performance.	Weiss and Ray (2019)	(i) Nature of trait: unspecified versus phenotypic versus soft, morphological, behavioural versus morphological, biochemical, physiological, structural, phenological, behavioural; (ii) cause (nature) of response: abiotic versus biotic and abiotic.
	Any trait which impacts fitness indirectly via its effects on growth, reproduction, and survival.	Violle et al. (2007)	
	Response traits influence the abilities of species to colonise or thrive in a habitat and to persist in the face of environmental changes.	Nock et al. (2016)	
	Response groups are identified through community‐level studies of changes in soft, morphological, or behavioural traits in response to abiotic or biotic factors.	Lavorel and Garnier (2002)	
	The characteristics of an organism that are considered relevant to its response to the environment and/or its effects on ecosystem functioning.	Díaz and Cabido (2001)	
	Morphological, biochemical, physiological, structural, phenological, or behavioural characteristics that are expressed in phenotypes of individual organisms and are considered relevant to the response of such organisms to the environment.	Díaz et al. (2013)	
Effect trait	Effect traits influence the fitness and/or organismal performance of an interacting partner and/or have an effect upon the environment.	Lavorel and Garnier (2002); Weiss and Ray (2019)	(i) Level of measurement: organism versus species; (ii) nature of affected entity: abiotic versus biotic and abiotic; (iii) nature of trait‐affected abiotic entity: (a) general (environment) versus specific, the latter, in turn, consisting of ecosystem (b) functioning versus processes versus properties (no definitions provided); (iv) nature of traits: unspecified versus physiological (harder) versus physiological + morphological, biochemical, structural, phenological, behavioural; (v) trigger: unspecified versus fitness expression.
	Organismal traits that influence ecosystem functioning.	Tilman (2001)	
	Components of an organism's phenotype that influence ecosystem level processes.	Petchey and Gaston (2006)	
	Those traits that determine a species' influence on ecosystem properties and, in turn, the services or disservices that human societies derive from them.	Nock et al. (2016)	
	Morphological, biochemical, physiological, structural, phenological, or behavioural characteristics that are expressed in phenotypes of individual organisms and are considered relevant to the effects of such organisms on ecosystem properties.	Díaz et al. (2013)	
	The characteristics of an organism that are considered relevant to its effects on ecosystem functioning.	Díaz and Cabido (2001); Campanyà‐Llovet et al. (2023)	
	Physiological, harder traits at the individual level.	Lavorel and Garnier (2002)	
	Traits describing the effects of an organism on habitat properties as a result of fitness expression, such as physiological activity, space occupation, moving, foraging, or sheltering.	Beauchard (2023)	

The concept of ‘trait’ is not the sole subject of debate. According to the prevalent definitions, for a trait to be ‘functional’, it ought to affect an organism's fitness or performance (McGill et al. 2006; Violle et al. 2007; Mouillot et al. 2013). In this regard, it is worth noting that, from an evolutionary viewpoint, if a trait bore no relevance to or influence on organismal fitness, it would not show in the organism's life history or ecology (Volaire et al. 2020; Sobral 2021; Streit and Bellwood 2023). Moreover, as traits are related to fitness depending on environmental variation—the latter encompassing coexisting species, resources, and abiotic factors—it has been argued that any trait could plausibly be related to organismal fitness in at least one potential environment (Sobral 2021). For instance, strong selection in favour of root traits would occur in a given plant population during a drought year, while the same population would experience strong selection in favour of flower traits during a year with severely restricted pollinator availability. Nonetheless, if the same plant population was to be investigated at any point in time with optimum precipitation levels and pollinator abundance, neither root nor flower traits could be realistically regarded as bearing no relevance to organismal fitness (Sobral 2021). If a potential connection exists between the spatiotemporal distribution of trait values and fitness in any of the possible environments, then all traits have the potential to affect organismal fitness and are, therefore, functional by definition (Volaire et al. 2020; Sobral 2021). Lastly, the well‐established practical difficulty associated with the documentation of trait–fitness relationships warrants a criterion for the distinction of ‘functional’ and ‘non‐functional’ traits that is independent of a trait's relationship to organismal fitness (Shipley et al. 2016; Volaire et al. 2020). Thus, it is unsurprising that, when asked about the conditions that a trait must fulfil to be regarded as ‘functional’, respondents to the Dawson et al.'s (2021) survey held diverging opinions, with ‘affect ecosystem processes’ (50% agree), ‘be related to resource acquisition’ (50% agree), and ‘define important niche dimensions’ (50% agree) emerging as particularly divisive. Moreover, despite several definitions stating it as a clear requirement (e.g., McGill et al. 2006; Violle et al. 2007), roughly a quarter of survey respondents did not believe that traits should affect organismal fitness in order to qualify as functional (Dawson et al. 2021). This, alongside the fact that the term ‘functional trait’ appears to be used in plant‐centric trait papers much more frequently than in their animal‐focused counterparts (Dawson et al. 2021), suggests that unification and clarity in the use of trait terminology may have been attained in plant ecology following the widely accepted definition by Violle et al. (2007), but that universally relevant terms that are applicable beyond the realm of vegetation remain unavailable. The fact that relatively recent papers continue to advance altered definitions of functionality and/or trait concepts (Bellwood et al. 2019; Dawson et al. 2019) appears to support the assertion that practitioners are unable to apply classic definitions to their respective fields of research. Based on Table 1, it could be argued that a divide appears to exist between researchers looking inwards and using traits to consider organism‐level fitness and those looking outwards and using traits to consider organisms' influence on their environment. To exploit the full potential of trait‐based approaches, clarification and agreement within the scientific community are required for the unification of future definitions, as no consistent operationalisation of trait research will ever be attained without first addressing *what* constitutes a trait and *how* traits are to be used.

### 3.2 How Are Traits Classified?

To support the selection of traits within and across taxa, an unambiguous classification system is required. However, much like trait terminology, the categorisation of traits suffers from a lack of standardisation (Table 2).

**TABLE 2:** Examples of trait classification frameworks adopted in trait‐based literature.

Framework	Focus/Classification	Source(s)
Response–effect	Direct connections: response traits are a direct response to external pressures, while effect traits exert a direct and measurable effect on ecosystem functioning.	Lavorel and Garnier (2002); Suding et al. (2008)
Pattern–process	Spatial versus temporal: pattern traits are measured irrespective of time (static) and along spatial gradients, while process traits are measured under environmental conditions fluctuating in time which characterise processes, that is, flows of material and energy in a given environment during a defined period of time.	Volaire et al. (2020)
M–P–P	Type over function: traits are classified with regard to their type, that is, what they relate to (morphology, physiology, phenology), rather than with respect to their ‘role’.	Violle et al. (2007)
**Same focus as M‐P–P, but different trait classification**		
M–P–P–E	Morphological–physiological–phenological–ecological performance (M–P–P–E)	Fountain‐Jones et al. (2015)
L–M–P–B	Life‐history–morphological–physiological–behavioural/mobility (L–M–P–B)	Litchman et al. (2013)
L–M–M–P–E	Life‐history–mobility–morphological–physiological–ecological (L–M–M–P–E)	Desrosiers et al. (2019)
L–B–M	Life‐history–behavioural–morphological (L–B–M)	Breine et al. (2018); Campanyà‐Llovet et al. (2023)

**FIGURE 2:**
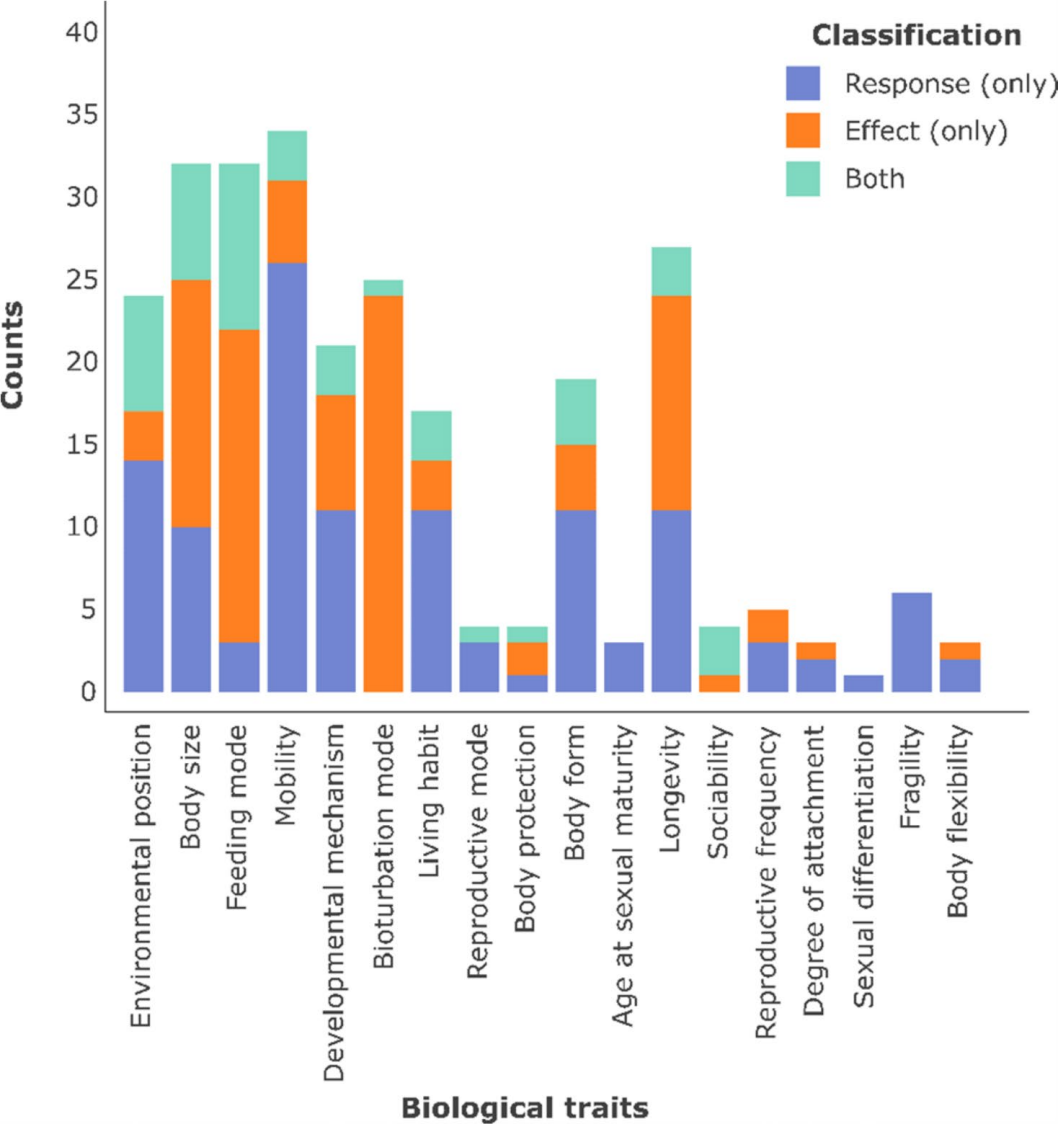
Inconsistency in trait classification within the response–effect framework. This framework was selected as an example owing to its consistent description across studies and a larger sample size (*n* = 45; pattern–process = 0; L–M–B < 10). Eight further articles adhered to the response–effect framework but failed to assign the investigated traits to either category, directly or indirectly through a description of the trait and its function(s). The traits reported were identified as common in benthic research following a literature search (see Section 2 and Figure 1).

The response–effect framework, first advanced by Lavorel and Garnier (2002), is the most widely adopted approach for trait classification across the body of trait literature. In the context of benthic trait research, an assessment—as part of the present work—of a sample of 398 published studies alongside the Biological Traits Information Catalogue (BIOTIC) revealed adherence to the response–effect framework by 13.3% of the articles, of which 5.7% appeared to further adopt the L–B–M classification system (Table 2). The L–B–M classification framework, or analogous approaches relying on type‐based categorisation, was found in 3.8% of the assessed articles. The majority of the investigated articles, 83.75%—in addition to the BIOTIC database—failed to provide information on the specific classification framework adopted by the authors. When considering trait classification with respect to a single framework, further inconsistencies emerged across studies (Figure 2).

**FIGURE 3:**
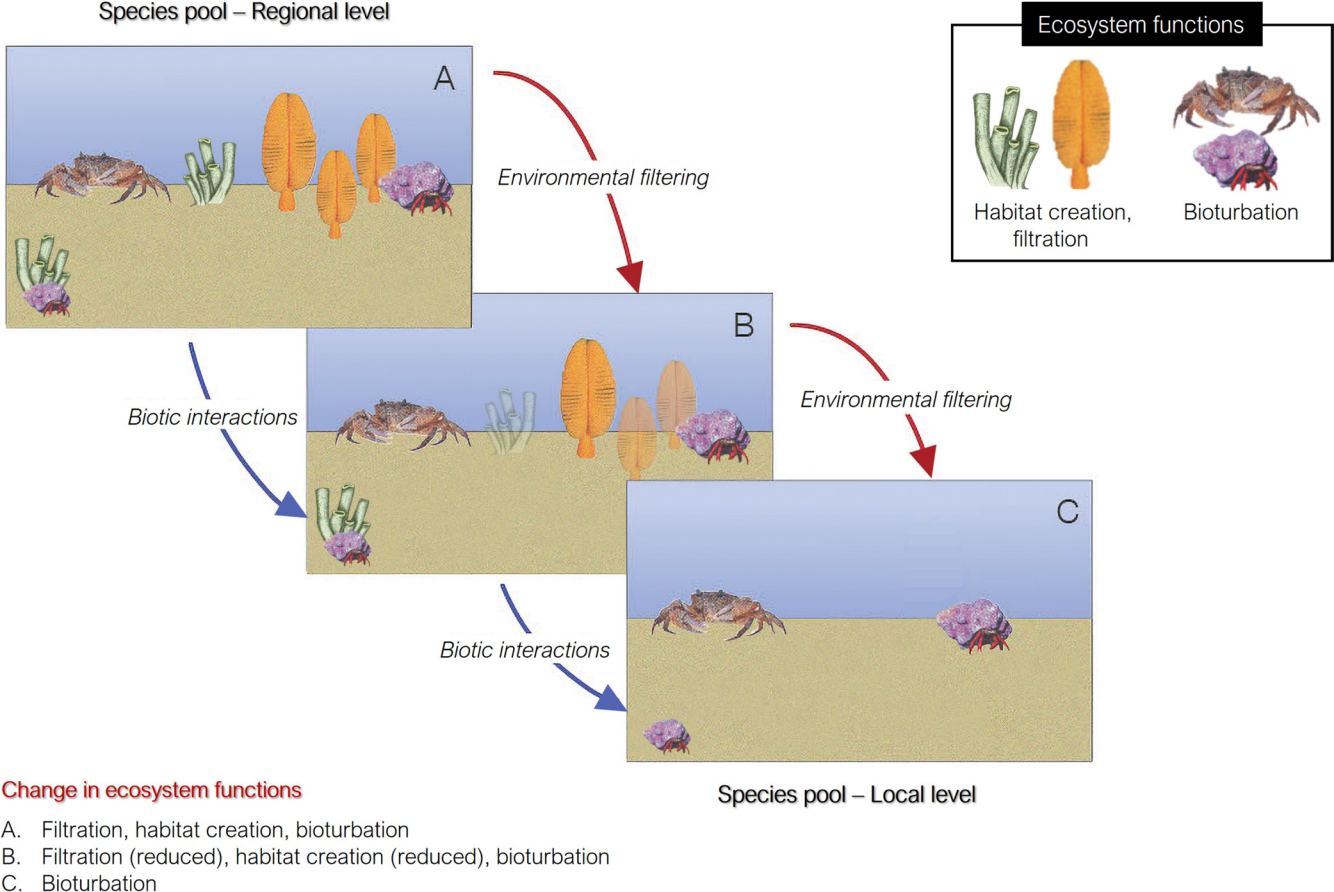
Schematic illustration of how abiotic and biotic environmental filtering may affect ecosystem function through changes to community dynamics and composition, from the regional species pool to local assemblages.

As the name suggests, the response–effect framework categorises traits into response and effect classes, the former encompassing attributes that describe the response of organisms to external perturbations, and the latter encompassing attributes that describe the influence of organisms on one or several ecosystem functions (sensu Bellwood et al. 2019). While the distinction between response and effect has long been implemented in the field of ecology to define competitive dynamics (e.g., Goldberg 1990), its application to ecosystem function only dates back two decades (e.g., Walker et al. 1999; Lavorel and Garnier 2002; Suding et al. 2008). The central tenet of the response–effect framework is that the structure and composition of a given community are the result of environmental filtering and biotic interactions excluding all phenotypes that do not possess appropriate response trait values from the regional species pool. These response traits may comprise responses to external perturbations that are direct and/or occurring through compensatory dynamics following alterations to species interactions (Suding et al. 2008). The altered community, in turn, influences ecosystem functioning via changes to the representation of effect traits (Suding et al. 2008; Garnier et al. 2016; Figure 3). The extent to which organisms with response traits favoured by the environmental filtering processes differ in their effect trait portfolio relative to the original assemblage is what determines the degree to which community change affects ecosystem function. Although it has been argued that a trait should contribute almost exclusively to either the response of an organism to external pressures or to its effect on the ecosystem (e.g., Suding et al. 2008), several traits have been demonstrated to relate strongly to both response and effect classes (e.g., Chalcraft and Resetarits Jr. 2003; Schmitz 2004). For example, the arborescent form of some coral taxa exerts important effects on other species through the creation of structurally complex biogenic habitats; however, the complexity of coral morphology is further indicative of the species vulnerability to physical disturbance, thus expressing survival potential (Darling et al. 2012). Therefore, the relationship between response and effect likely depends on the specific traits considered (Suding et al. 2008).

**FIGURE 4:**
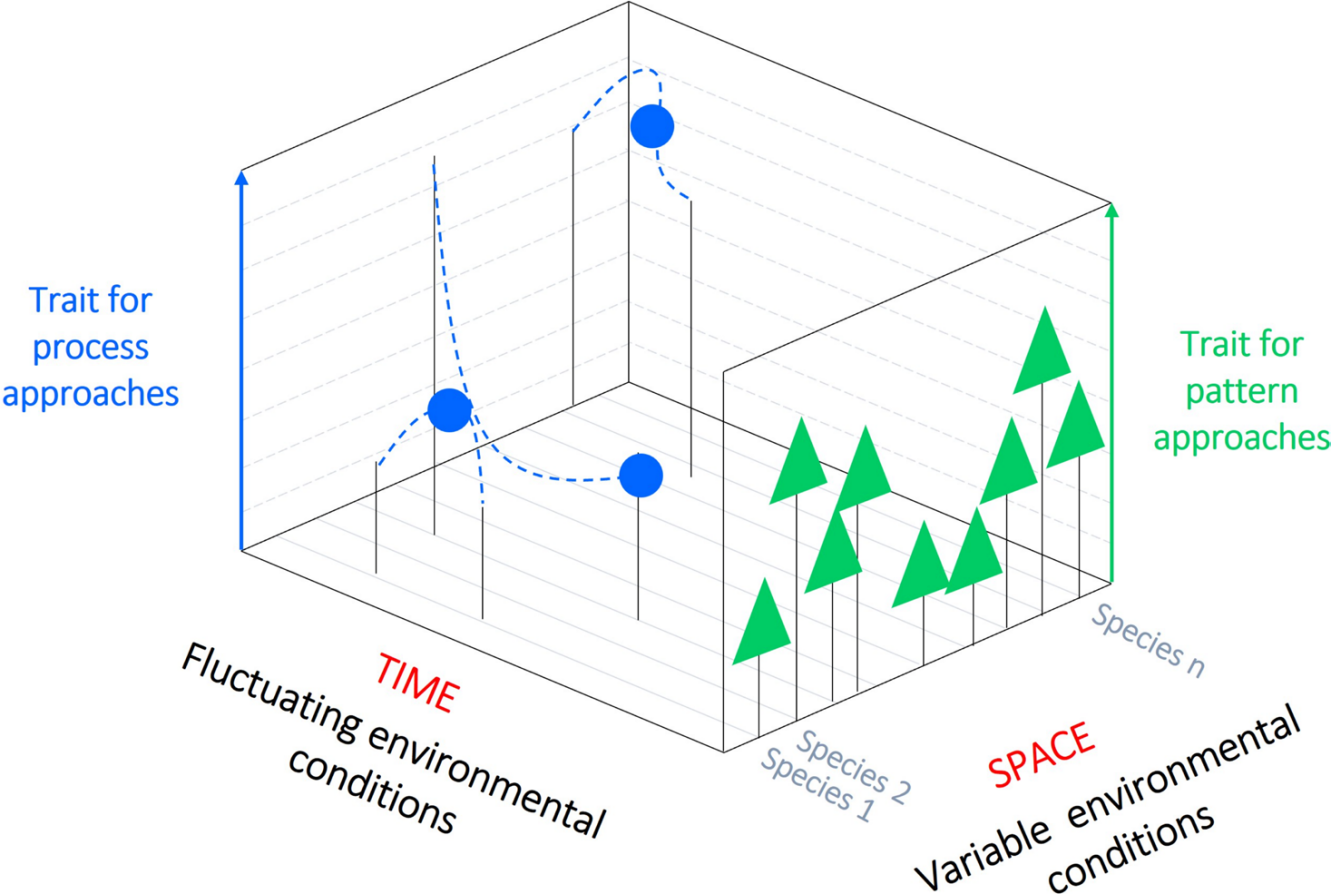
Schematic illustration of traits measured across individuals of different taxa (and/or populations). Pattern traits (green triangles) account for variability of individual functioning at a given time and across spatial gradients/environmental conditions; process traits (blue circles) account for variability of individuals under fluctuating environmental conditions and across a relevant period. The representation of process traits (blue) shows values (e.g., slope, minimum, maximum, inflexion point) of mathematical functions established between a biological response and time or environmental variation (blue dashed line). Adapted from Volaire et al. (2020).

The pattern–process trait classification framework was proposed by Volaire et al. (2020) in the context of plant ecology to promote more explicit connections between trait‐based ecology and other disciplines. It has its roots in the understanding of observed patterns with respect to the processes that engender them (Levin 1992). Although by no means novel in the scientific discourse, the distinction between patterns and processes has, thus far, been scarcely adopted for ecological purposes (Levin 1992). According to the pattern–process approach, traits fall under two distinct, yet complementary, categories depending on whether they are assessed as a function of time. Traits that are measured in standardised, comparable—and generally optimum—conditions, irrespective of time, are classed as ‘pattern’ traits and are said to afford a synchronic snapshot of the observed variability at any given time (Figure 4). Such traits are predominantly morphological, structural, or physiological in nature, shaped by evolutionary processes and accounting for a substantial share of the variation in resource acquisition and/or resource conservation. Conversely, traits that are measured under temporally fluctuating abiotic conditions and which characterise processes are referred to as ‘process’ traits—‘processes’ are defined herein as the flow of energy and material in a particular environment during a well‐defined temporal window. Process traits are generally measured as the response to temporally dynamic abiotic factors and need to be measured as a function of time as well as repeatedly over time (Figure 4).

From a conceptual viewpoint, pattern traits allow for observed structures and patterns within a given community to be ascertained across resource gradients, irrespective of their origin (Reich 2014; Volaire et al. 2020). On the other hand, the utilisation of process traits is recommended when the scope of the research objectives concerns resource acquisition and expenditure mechanisms—particularly for metabolic, mobility, growth, reproductive, and survival purposes—as well as when focusing on the mechanisms underpinning resource cycling, storage, and/or loss through biotic and abiotic processes at the ecosystem scale (Volaire et al. 2020). The pattern–process trait classification framework holds broad relevance for any trait‐based approach, irrespective of the biological model (Volaire et al. 2020). Particularly with respect to benthic systems, a classification framework that accounts for the spatiotemporal variability inherent to benthic species may favour a shift away from a rather static trait analysis approach (de Juan et al. 2022). While some traits might be largely static, for example, morphology, others, such as burrowing behaviour, may experience substantial variability over an individual's life span. The use of average trait values—a common approach in traditional trait‐based ecology—could bias predictions of both responses to external disturbances and effects on ecosystem functions (de Juan et al. 2022). Such spatiotemporal variability must be better comprehended if accurate models of biological trait–ecosystem functioning are to be developed.

A further trait classification framework that has emerged as relatively common from the reviewed body of literature is the morphological–physiological–phenological (M–P–P) approach, first advanced by Violle et al. (2007) in the context of plant ecology and successively adapted to organisms and systems beyond terrestrial vegetation (Table 2). The M–P–P framework hinges on the fact that the performance of a species in a particular ecological context depends on the ability of individual organisms to grow, reproduce, and survive (i.e., Darwinian fitness). In living organisms, this ability is assessed through the quantification of components of whole organism performance (e.g., body size, age at sexual maturity; Le Galliard et al. 2004), such components being referred to as ‘performance traits’. This particular choice of terminology is in reference to the ‘morphology, performance, and fitness’ paradigm proposed by Arnold (1983) for the study of animal taxa. According to this paradigm, morphological traits affect (directly and indirectly) performance traits, which, in turn, affect fitness, either directly or indirectly. It is, therefore, argued that the value of performance traits is dictated by morphological, physiological, and phenological traits operating from the cell to the whole organism. A classification framework that distinguishes traits by type according to their biological field of relevance (morphology, physiology, and phenology) has been argued to bare potential to stimulate a search for general patterns, forgoing the confusion that may arise from the inconsistent use of terminology (Litchman et al. 2013). However, whilst advocating what is, arguably, a more rigorous classification strategy, it is noteworthy that a vast portion of research implementing the M–P–P framework (or a revisited version of it) still relies on the concepts of ‘response’ and ‘effect’ traits (e.g., Violle et al. 2007; Fountain‐Jones et al. 2015).

### 3.3 How Many Is Too Many? Trait Synonyms in Benthic Ecology

**FIGURE 5:**
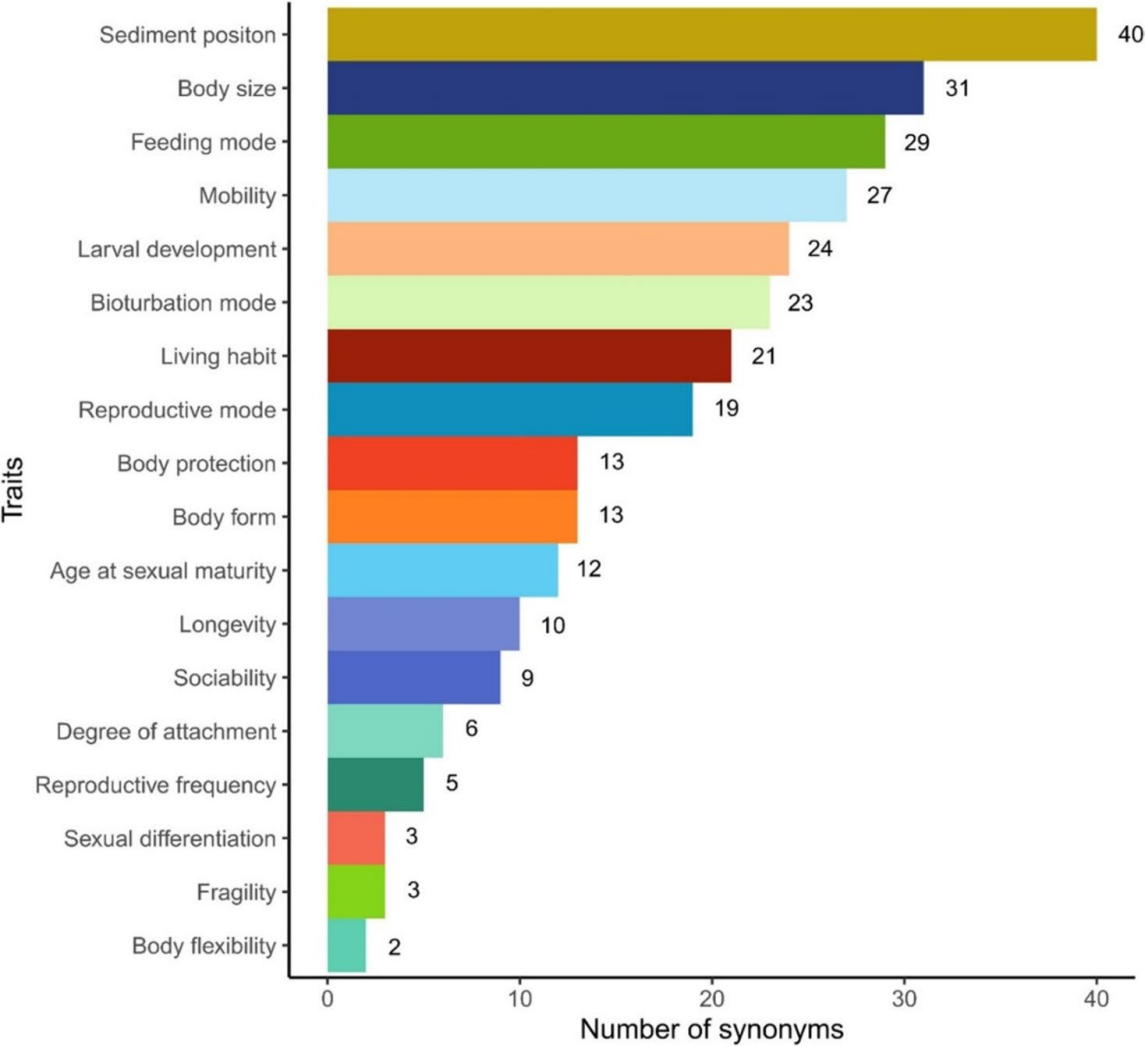
Bar chart of the synonym distribution for 18 commonly implemented traits identified in a sample of published benthic trait literature (*n* = 407).

The lack of standards in terminology has long been recognised as a major barrier to the synthesis of trait knowledge, prompting numerous publications on the subject (e.g., Schneider et al. 2019; Weiss and Ray 2019; Gallagher et al. 2020; Lam‐Gordillo et al. 2020a, 2020b; Dawson et al. 2021; de Juan et al. 2022). As previously illustrated, the absence of widely accepted and unambiguous trait definitions applicable to a diverse range of study organisms has led to a proliferation of alternative designations (e.g., Bellwood et al. 2019; Dawson et al. 2019, 2021), with a growing number of classification frameworks fuelling further confusion. This, however, does not represent the full extent of the issue. Trait nomenclature is a remarkably fertile source of inconsistencies. Across the 407 articles (and BIOTIC database) that made up the sample for this assessment, 18 traits commonly used in benthic research were found to be named inconsistently (Table S1). Each was found to be associated with a minimum of two and a maximum of 40 synonyms (Figure 5), amounting to a total of 290 and an average of 16 synonyms per trait (16.1 ± 11.15 SD). The proliferation of synonyms often reflects the representation of a particular trait within the literature; for example, body flexibility is associated with two synonyms and only features in 10 articles. However, other traits exhibit more synonyms despite enjoying a comparable representation; for example, age at sexual maturity is associated with 12 synonyms and is discussed in 11 articles. Similarly, although environmental position features in 58 articles, it is associated with nearly twice as many synonyms as mobility, which features in 75 articles.

Equally, the reporting of modalities (i.e., values, numerical or otherwise) within traits was found to exhibit substantial cross‐study variation. Firstly, the modalities reported for a given trait were found to differ across studies, despite all works focusing on benthos. Secondly, different wordings were detected in reference to the same underlying phenomenon/concept (Table S1). Examples include the arbitrary addition of different suffixes (e.g., crawl‐er/−ing, attach‐ed/−ment, omnivor‐e/−y/−ous), intensifiers (e.g., very/highly fragile), and/or descriptive terms (e.g., active/passive suspension feeding), as well as different spellings of compound words (e.g., semi‐/semicontinuous). Moreover, what is presented here as a trait modality (as in Foveau and Dauvin 2017; Boyé et al. 2019; Lam‐Gordillo et al. 2020b) was, at times, referred to as a ‘trait category’ (e.g., Oug et al. 2012; Donadi et al. 2015; Clare et al. 2022; Shi et al. 2023), with ‘trait category’ described elsewhere as an ensemble of traits (e.g., Tyler et al. 2012; Clark et al. 2016; Gravel et al. 2016; Chapman et al. 2019).

Lack of standardisation in this remit of trait research has attracted limited attention (but see Chapman et al. 2019; Degen and Faulwetter 2019; Lam‐Gordillo et al. 2020a, 2020b). Adoption of ad hoc naming conventions represents a major barrier to the interoperability (sensu Wilkinson et al. 2016) of trait data, resulting in data that are not machine‐readable, thus hindering cross‐study comparability and automated data extraction (Wilkinson et al. 2016; Schneider et al. 2019; Sansone et al. 2022). For example, a search of multiple information systems using one of the names reported for any given trait in the literature would likely fail to return data associated with its synonyms, despite their shared meaning. By selecting the ‘wrong’ keyword, researchers risk missing large sets of relevant data, possibly resulting in erroneous conclusions (Lam‐Gordillo et al. 2020a). As a lack of standardisation in the reporting of trait data exacerbates data heterogeneity and contributes to the decentralisation of the field, inconsistent vocabulary use may also lead to duplication of effort (Gallagher et al. 2020). With a growing number of small, isolated, and heterogeneous data sources exacerbating the disconnect between pools of trait data and limiting knowledge sharing across taxa and ecosystems (Farley et al. 2018), researchers are more likely to collect redundant data and develop overlapping tools for data collation, cleaning, and integration (Gallagher et al. 2020). As these small, isolated repositories continue to grow, difficulties with data integration and synthesis will also increase due to the momentum of entrenched workflows and exchange protocols that may not be interoperable (Gallagher et al. 2020).

## 4 Where Do We Go From Here and Why Does It Matter?

Marine biological traits (e.g., downward conveying abilities) can be linked to ecosystem services (e.g., carbon sequestration) which provide benefits to humans (e.g., climate regulation). Understanding which biological traits are involved in the delivery of which ecosystem services can help identify management options that minimise undesired trade‐offs between seemingly diverging ecosystem service demands (Hanisch et al. 2020). Identification of links between services and biological traits may further support the detection of potential synergies (sensu Hanisch et al. 2020) among services, that is, the possibility to simultaneously support multiple ecosystem services by supporting communities with traits involved in their delivery. The study of biological traits can contribute to marine spatial planning and the development of ecological risk assessments (ERAs) for regulated anthropogenic activities (e.g., deep‐sea mining, Boschen‐Rose et al. 2021), affording a community‐ or assemblage‐level appreciation of functional sensitivity (sensu organismal functioning). The use of biological traits thus allows for spatial patterns of organismal sensitivity to disturbance to be obtained (i) across a vast array of sites and habitats with different species compositions (Hewitt et al. 2008) or (ii) under circumstances where detailed species‐specific knowledge is missing (Tyler‐Walters et al. 2009; Hewitt et al. 2022). Despite the manifold benefits of a trait‐inclusive management approach, managers have failed to embrace the use of biological traits in marine conservation practices (Miatta et al. 2021). Such reluctance possibly reflects, among others, a lack of accessible information (see Section 3.3) and standardised protocols detailing the use of traits for conservation purposes. Ambiguous terminology hinders the generalisation of both trait data and outputs, severely limiting their relevance to conservation interventions (de Juan et al. 2022).

As a first step towards the unification of benthic trait research, we propose the following guidelines aimed at standardising studies for policy and management applications. Firstly, with regard to definitions, we encourage the research community to always specify which trait definition is being used and why a given definition is being favoured over others, so as to minimise confusion and maximise transparency. Secondly, in agreement with Streit and Bellwood (2023), we further encourage the scientific community to abandon the term ‘functional’ when referring to organismal traits in favour of the more general term ‘biological trait’. Four reasons lie behind such a recommendation. First, as discussed earlier in this review, any trait could plausibly be related to organismal fitness in at least one potential environment (Sobral 2021), meaning that all traits are, by definition, functional. If the concept of functionality is taken to encompass all traits affecting the fitness of a living organism (e.g., McGill et al. 2006; Violle et al. 2007), then the use of the word ‘functional’ as a modifier of the word ‘trait’ becomes redundant. Second, trait–fitness relationships are difficult to establish practically, and, without a criterion for the distinction of ‘functional’ and ‘non‐functional’ traits that is independent of a trait's relationship with organismal fitness, the classification of a trait as ‘functional’ is, arguably, arbitrary (Shipley et al. 2016; Volaire et al. 2020). Thirdly, likely as a result of the above, the term ‘functional’ in reference to the nature of a trait has long been the cause of substantial confusion, with benthic researchers using it to refer to properties affecting ecosystem processes, related to resource acquisition, and/or defining important niche dimensions (see Dawson et al. 2021). Moreover, while several definitions state it as a clear requirement, some researchers do not believe that traits should affect organismal fitness in order to qualify as functional (Dawson et al. 2021). Given the lack of agreement within the scientific community around what constitutes a ‘functional trait’, the term ‘biological trait’ offers a more general and scientifically accurate alternative that is likely to appease the different fields of benthic research. A trait, as per all the available definitions (Table 1), is, at the very least, a quantifiable property of a living organism, making it biological by nature. Like the field of biology, a biological trait can then be divided into specialised forms reflective of an organism's morphology, physiology, anatomy, behaviour, origin, and distribution. Lastly, within the ecosystem service narrative, ecosystem functioning—which gives rise to ecosystem services—is often described as ‘the *biological*, physical, and geochemical processes that occur within an ecosystem and consider the sizes of compartments (such as carbon storage) as well as the rates of their changes (such as rates of carbon sequestration)’ (Hooper et al. 2005). By making the change to ‘biological’ traits, a distinction between the role of biology in ecosystem function and processes (Zhang et al. 2022) and the pathways to ecosystem services is being made, possibly better informing conservation planning and management.

Thirdly, considering its successful integration in trait ecology research—both terrestrial and marine—we strongly encourage the scientific community to ‘universally’ adopt the response–effect framework when aiming to influence the political sphere (see Section 3.2). This aligns research with requirements to develop and implement policy and associated management decisions and tools. The response–effect framework is used implicitly in projects commissioned by UK statutory nature conservation agencies to support their work. An example includes the structured sensitivity assessment methods developed by the Marine Life Information Network (MarLIN, Tyler‐Walters et al. 2023) which consider response traits in the assessment of pressure impacts, an approach that has been subsequently extended to both the creation of trait catalogues (MarLIN 2006) and the definition of ecological groups and their sensitivity to pressures (Tillin and Tyler‐Walters 2014a, 2014b). The response–effect framework has further been used in the development of conceptual ecosystem models of the marine environment incorporating the benthos to identify indicators (e.g., Alexander et al. 2014; Hinchen et al. 2021) and assess the provision of ecosystem services (effect traits) as well as the sensitivity of these (response traits) to human activities (Tillin et al. 2020). Additionally, the framework has been applied to the development of protocols to evaluate designation boundaries for Special Areas of Conservation, SACs (European Habitats Directive; Bremner et al. 2006), and to the development of maps reflecting spatial variations in marine benthic trait expression (Bolam et al. 2023). Such maps support licensing decisions and marine spatial planning by identifying regions of expected high tolerance, on the part of benthic assemblages, to a given pressure, as well as areas of elevated (potential) ecological function that should be protected.

**FIGURE 6:**
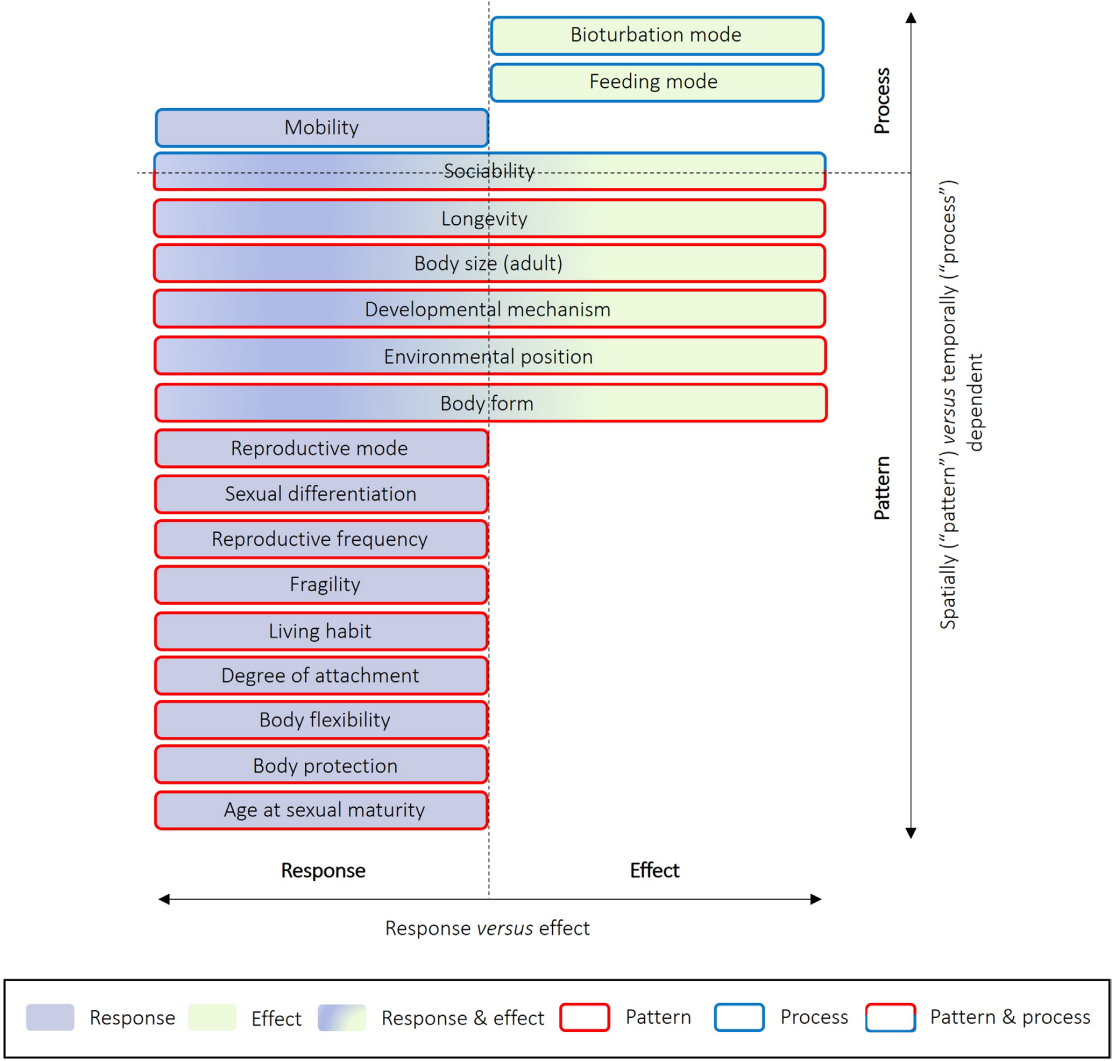
Recommended naming of the 18 traits assessed in this review with their respective recommended classification for policy and management purposes, integrating the seminal and intuitive response–effect trait classification framework with the more recent pattern–process framework.

Fourthly, given the importance of both spatial and temporal dimensions for effective marine spatial planning (Shucksmith and Kelly 2014), we encourage the scientific community to integrate, whenever possible, the response–effect framework with its pattern–process counterpart, as shown in Figure 6 with respect to the 18 traits assessed in this review. Traditional benthic trait analysis has been described as ‘static’, focusing on average trait values and disregarding the spatial and temporal variabilities that characterise benthic trait expression (de Juan et al. 2022). Biological traits may vary ontogenetically (e.g., larvae may disperse over long distances and become sedentary once attached and in the adult stage), but also over shorter time scales (e.g., diurnal changes in burrowing activities). By drawing on both the response–effect and the pattern–process frameworks, our suggested classification approach not only reflects the predominant convention in both benthic trait research and the policy realm, but it also offers a flexible and adaptable framework which is likely to satisfy a range of different research questions. For a detailed justification of the reported trait classifications (Figure 6), the reader is directed to the Appendix S1 (Section S1) at the end of this review.

Lastly, regarding naming conventions, we ask the scientific community not to further proliferate on the terms assembled in Table S1 and to use the terminology adopted by this review (see Section 2). This is because, when reporting traits in this article, the decision was made to refer to traits by their most adopted name in the literature, so as to ‘accelerate’ terminology standardisation. The trait names reported here, therefore, do not reflect our personal ‘favourites’, but rather the naming conventions already adopted by the vast majority of benthic researchers. Three exceptions were made. In the case of ‘developmental mechanism’ and ‘environmental position’, we encourage the research community to adhere to the BIOTIC nomenclature over the more common ‘larval development’ and ‘sediment position’, respectively. This is because not all species present a larval stage and not all species inhabit the sediment. Similarly, despite ‘life span’ (or a version of it) being more common in the reviewed literature, the one‐word term ‘longevity’ was chosen over the two‐word counterpart to reduce inconsistencies (e.g., ‘life span’, ‘lifespan’, ‘life‐span’, see Table S1).

However, recognising that numerous other standards exist which may have been used in published research over the past two decades, we propose a ‘translation’ table (Table 3) intended for use by trait ecologists when reviewing existing literature. The latter may classify traits of interest according to the L–M–B classification framework or a revisited version of it, which we do not recommend for use in the policy and management realm. Such frameworks fail to provide decision makers with immediate knowledge of why a given trait bears relevance to species/habitat/ecosystem service protection, as they merely indicate the branch of biology to which a trait relates. Moreover, research implementing the L–M–B framework often still relies on the concepts of ‘response’ and ‘effect’ traits (e.g., Violle et al. 2007; Fountain‐Jones et al. 2015). As shown in Figure 2, substantial inconsistency exists in how traits are classified even within a single framework, with a harmonised process thus being warranted. The following table has been produced following an extensive review of the literature, with references provided throughout our list of justifications (Appendix S1, Section S1). Therefore, the proposed classification reflects up‐to‐date evidence and conventions.

**TABLE 3:** Translation table for the reporting and classification of the 18 traits discussed in this review.

Traits	Response–effect		Pattern–process		Life history–morphology–behavioural		
	R	E	Pa	Pr	L	M	B
Longevity	X	X	X		X		
Body size	X	X	X			X	
Mobility	X			X			X
Feeding mode		X		X			X
Reproductive mode	X		X		X		
Sexual differentiation	X		X		X		
Reproductive frequency	X		X		X		
Fragility	X		X			X	
Developmental mechanism	X	X	X		X		
Living habit	X		X				X
Bioturbation mode		X		X			X
Environmental position	X	X	X				X
Degree of attachment	X		X			X	
Body flexibility	X		X			X	
Sociability	X	X	X	X			X
Body protection	X		X			X	
Age at sexual maturity	X		X		X		
Body form	X	X	X			X	

We suggest that future reviews consider the use of the trait‐based approach in benthic research from a methodological perspective, describing the approaches currently available for the acquisition of trait data (continuous, discrete, and qualitative) and the quantification of trait diversity. For qualitative and discrete traits in particular, harmonised protocols would be beneficial and would represent an important step forward towards the operationalisation of the trait‐based approach.

## 5 Conclusions

This review affords a synthesis of the inconsistencies permeating benthic trait literature and, more broadly, trait research as a whole. A plethora of often diverging definitions of ‘trait’ and associated concepts are identified, with the concept of ‘functional trait’ being particularly divisive. Moreover, a total of three distinct classification frameworks are identified, all having originated in the context of plant ecology and two already featuring in benthic trait research, albeit to different extents. Trait nomenclature is also a fertile source of inconsistencies, with an average of 16 synonyms per trait determined for 18 traits commonly featuring in benthic literature. The reporting of trait modalities also exhibits substantial cross‐study variation, with discrepancies often deriving from the arbitrary addition of a diverse array of suffixes, intensifiers, or descriptive terms.

Over the last two decades, the advent of trait‐based approaches has undoubtedly enriched our understanding of key ecological processes, with biological traits proving to be critical in elucidating how the environment affects, or is affected by, different organisms. Biological traits further hold untapped potential with regard to marine environmental management, their analysis facilitating a spatial understanding of ecosystem functions and services to inform decision making. Despite the obvious benefits of a trait‐inclusive conservation approach, managers have thus far failed to embrace the use of biological traits in marine spatial planning. Such reluctance possibly reflects, at least partly, a lack of standardised protocols and accessible information on many marine species. As a first step towards the unification of benthic trait research, we propose the following guidelines aimed at standardising studies for marine policy and management applications:

- We encourage the scientific community to always specify the trait definition which is being used and the reason(s) behind the selection, so as to minimise confusion and maximise transparency;
- We encourage the scientific community to abandon the term ‘functional’ when referring to organismal traits in favour of the more general and scientifically accurate term ‘biological trait’;
- When aiming to influence the political sphere, we urge the scientific community to adopt the response–effect trait classification framework because of its successful integration in trait ecology research (both terrestrial and marine), as well as its intuitive and binary nature;
- Whenever possible, we encourage the scientific community to integrate the response–effect framework with the pattern–process framework, the latter holding potential for incorporating the spatial and temporal variabilities of trait expression in benthic trait research;
- When aiming to influence the political sphere and reviewing literature which classifies traits of interest according to the L–M–B classification framework or analogous approaches, we ask that the scientific community consult our ‘translation’ table (Table 3) to avoid further exacerbating the inconsistent classification of benthic traits (see Section 3.2); and,
- Lastly, we encourage the scientific community not to further proliferate on the trait naming conventions and to use the terminology adopted in this review, as it reflects the most adopted trait names in the literature, thus ‘accelerating’ terminology standardisation.

Future reviews should explore the use of the trait‐based approach in benthic research from a methodological perspective, as harmonised protocols for trait data acquisition and trait diversity quantification would represent an important step forward towards the full operationalisation of the trait‐based approach.

## Author Contributions

**Irene Susini:** conceptualization (equal), data curation (lead), formal analysis (lead), investigation (lead), methodology (equal), resources (lead), visualization (lead), writing – original draft (lead), writing – review and editing (lead). **Heidi M. Tillin:** resources (supporting), supervision (supporting), writing – review and editing (equal). **Louise Anderson:** resources (supporting), supervision (supporting), writing – review and editing (equal). **Craig M. Robertson:** methodology (equal), resources (equal), supervision (supporting). **Sian Rees:** methodology (equal), supervision (supporting), writing – review and editing (equal). **Kerry L. Howell:** conceptualization (equal), funding acquisition (lead), project administration (lead), resources (supporting), supervision (lead), writing – review and editing (equal).

## Conflicts of Interest

The authors declare no conflicts of interest.

## Supporting information


Appendix S1

Data S1.

## Data Availability

All data are available within the manuscript, the Appendix S1, and the Supporting Information.
