# Supplementary material for: Towards Greater Standardisation in Benthic Trait Research to Support Application to Environmental Management
Source: Ecol Evol. 2025 Mar 4;15(3):e71072. doi: 10.1002/ece3.71072 (PMC11879613; doi:10.1002/ece3.71072)
Supplement: Supplementary file 1 — Appendix S1 [file ECE3-15-e71072-s002.docx]

## **Appendix**

## **Justification for trait classification**

**Response (R) traits:** attributes that describe the response of organisms to external perturbations (e.g., mobility, reproductive traits, fragility).

**Effect (E) traits:** attributes that describe the influence of organisms on one or several ecosystem functions (sensu Bellwood et al., 2019). Examples include longevity, body size, and bioturbation mode.

**Pattern (Pa) traits:** shaped by evolutionary processes and account for a substantial share of the variation in resource acquisition and/or resource conservation, contributing to the understanding of growth, fecundity, and survival differences. Such traits are predominantly morphological, structural, or physiological in nature (e.g., longevity, body size).

**Process (Pr) traits:** traits that are measured under temporally fluctuating abiotic conditions and which characterise processes, defined herein as the flow of energy and material in a particular environment during a well-defined temporal window. Process traits are generally measured as the response to temporally dynamic abiotic factors and need to be measured as a function of time as well as repeatedly over time.

**Life history (L) traits:** they pertain to life strategies resulting from evolutionary convergences which, in turn, result from universal energetic allocation trade-offs (Greenslade, 1983; Southwood, 1988; Kindsvater et al., 2016) (e.g., longevity, reproductive traits).

**Behavioural (B) traits**: traits which may vary over time as a result of both external factors, e.g., threats from other organisms, sound and olfactory stimuli, and internal stimuli, e.g., hunger (e.g., mobility, feeding mode, bioturbation mode).

**Morphological (M) traits:** traits pertaining to the form, shape, or structure of an organism (e.g., body size, body protection, body form).

**Longevity:** (**R–E**) this trait can be classified as ‘response’ as the length of an organism’s life span is an indication of how susceptible said organism is to disturbance, i.e., a long-lived, slow-growing organism is more vulnerable to disturbance than a short-lived, fast-growing organism as the recovery time is greater. Longevity can also be classified as ‘effect’ because it provides an indication of the relative investment of energy in somatic growth relative to reproduction; it can also be an indicator of the relative age of sexual maturity, that is, a proxy for an organism’s position in the *r* versus *K* strategy dichotomy. (**Pa**) Longevity is also an example of a ‘pattern’ trait, as, theoretically, it can be measured irrespective of time and affords a snapshot of the observed variability in the age structure of a community at any given time. (**L**) Lastly, longevity is an example of a ‘life history’ trait, as it pertains to a life property resulting from evolutionary convergences which, in turn, result from universal energetic allocation trade-offs (Greenslade, 1983; Southwood, 1988; Kindsvater et al., 2016).

**Body size:** (**R–E**) this trait can be classified as ‘response’ as the size of an organism provides an indication of how easily an organism can be impacted by some external pressures, i.e., a larger organism is more vulnerable to mechanical disturbance than a small organism which can easily find refuge. Body size can also be classified as an ‘effect’ trait as the size of an organism has implications with respect to the movement of Organic Matter (OM) in the benthic ecosystem. Large-bodied organisms hold OM longer within the benthic system, i.e., low turnover, relative to smaller sized counterparts which exhibit high turnover. (**Pa**) Body size is also an example of a ‘pattern’ trait. (**M**) Lastly, the body size of an organism is an expression of its morphology.

**Mobility:** (**R**) this trait is an example of a ‘response’ trait, as it offers an indication of how likely an organism is to escape external disturbance and relocate, if necessary, and also to recolonise post-perturbation from connected populations. Highly mobile organisms are more likely to survive and recover from external disturbance relative to low-mobility or sedentary organisms. (**Pr**) Mobility can also be classified as a ‘process’ trait, as this trait concerns an expenditure (energy) mechanism. To obtain a representative account of an organism’s mobility capacity, this trait should be measured as the response to temporally dynamic abiotic/biotic factors (i.e., as a function of time) as well as repeatedly over time. In fact, an organism may only exhibit a degree of mobility as a result of disturbance, with different disturbance levels eliciting different mobility responses. Mobility may also vary ontogenetically, therefore varying as a function of time. (**B**) Lastly, mobility is an example of a ‘behavioural’ trait as it may vary over time as a result of both external factors, e.g., threats from other organisms, sound and olfactory stimuli, and internal stimuli, e.g., hunger.

**Feeding mode:** (**E**) Feeding mode can be classified as an ‘effect’ trait as it bears important implications for the potential for transfer of carbon between the sediment matrix and overlying waters. This trait also has important repercussions for biogeochemical processes in the sediment. (**Pr**) Feeding mode is further example of a ‘process’ trait, as it relates to the flow of energy and material in a particular environment during a well-defined temporal window and concerns resource acquisition and expenditure (energy) mechanisms. It also may vary ontogenetically, therefore varying as a function of time. (**B**) Lastly, feeding mode is an example of a ‘behavioural’ trait as it may change over time and vary under different conditions.

**Reproductive mode, sexual differentiation, reproductive frequency, and developmental mechanism:** (**R**) these traits can be classified as ‘response’ traits as they afford an indication of the ability of recovery after a substantial reduction of population numbers (Costello et al., 2015). Organisms reproducing sexually (internal or external fertilisation, brooding) are more vulnerable to a reduction in organism numbers (e.g., allee effects) within a population than organisms that reproduce asexually, e.g., by fission. Similarly, gonochoristic organisms where the male and female reproductive organs are located in different organisms are more vulnerable to a sudden reduction in organism numbers within a population than hermaphroditic or asexual organisms, where offspring derives from a single parent organism. Organisms characterised by a high reproductive frequency (*r* strategists) are also more likely to recover following mechanical disturbance relative to organisms characterised by low reproductive frequency (*K* strategists). Lastly, egg-laying/brooding species inhabiting highly trawled areas are more likely to suffer substantial losses (i.e., parent plus offspring) due to the inability of the offspring to escape the area. (**E**) Larval development can further be classified as an ‘effect’ trait as it indicates the potential for dispersal of the larval stage prior to settlement. Larval development plays an important role in the export of carbon and energy, under the form of offspring, out of the system, and represents a critical factor in the effect of the community on ecosystem stability. (**Pa**) Owing to the fairly static nature of physiological attributes (Volaire et al., 2020), larval development can be regarded as a ‘pattern’ trait. (**L**) Lastly, reproductive mode, sexual differentiation, reproductive frequency, and larval development are all examples of ‘life history’ traits, as they pertain to life strategies resulting from evolutionary convergences which, in turn, result from universal energetic allocation trade-offs (Greenslade, 1983; Southwood, 1988; Kindsvater et al., 2016).

**Fragility:** (**R**) this trait can be classified as a ‘response’ trait as it provides an indication of the vulnerability of an organism to physical disturbance. A fragile, highly breakable organism is more prone to damage than a robust, hard-shelled organism. (**Pa**) Owing to the fairly static nature of morphological features (Volaire et al., 2020), fragility can be regarded as a ‘pattern’ trait. (**M**) Lastly, the fragility of an organism is an expression of its structural morphology.

**Living habit:** (**R**) this trait can be classified as a ‘response’ trait as it indicates the potential for the adult stage to evade or to be exposed to physical disturbance. Organisms differing in their living habitats will vary in their response to disturbance depending on their living habit, mobility, and sediment position. For example, burrow-dwelling organisms are more likely to evade disturbance relative to surface crawlers or epifaunal (attached) organisms. (**Pa**) Living habit can also be classified as a ‘pattern’ trait, as it describes a pattern in the structure of a community at any given time and can vary spatially along environmental gradients. (**B**) Lastly, living habit is an example of a ‘behavioural’ trait as it may vary over time as a result of both external, e.g., threats from other organisms, sound and olfactory stimuli, and internal stimuli, e.g., hunger.

**Bioturbation mode:** (**E**) this trait can be classified as an ‘effect’ trait as the sediment reworking ability of an organism plays an important role with respect to sediment–water exchange and sediment biogeochemical properties, thus exerting an effect on ecosystem functioning sensu transfer of energy and material through the system. (**Pr**) Bioturbation mode can also be classified as a ‘process’ trait as it relates to a mechanism underpinning resource cycling, storage, and/or loss through biotic and abiotic processes at the ecosystem scale (Volaire et al., 2020). Also, it may experience substantial variability over an individual’s life span, thus varying as a function of time. (**B**) Lastly, bioturbation mode is an example of a ‘behavioural’ trait as it may vary over time as a result of both external, e.g., threats from other organisms, sound and olfactory stimuli, and internal stimuli, e.g., hunger.

**Environmental position:** (**R–E**) this trait can be classified as a ‘response’ trait as it indicates the potential for the adult stage to evade or to be exposed to physical disturbance. For example, deep infaunal organisms (>5 cm depth) are more likely to evade disturbance than epifaunal or shallow infaunal (<5 cm) organisms. Sediment position can further be regarded as an ‘effect’ trait as it has repercussions for the surrounding environment. For example, benthopelagic species constitute a link between the sediment and the water column. Similarly, deep infaunal organisms affect sediment structure. (**Pa**) Sediment position can also be classified as a ‘pattern’ trait, as it describes a pattern in the structure of a community which can be measured irrespective of time and under optimised conditions (e.g., in laboratory settings) as a synchronic snapshot of the observed variability at any given time. (**B**) Lastly, sediment position is an example of a ‘behavioural’ trait as it may vary over time as a result of both external, e.g., threats from other organisms, sound and olfactory stimuli, and internal stimuli, e.g., hunger.

**Degree of attachment:** (**R**) this trait can be classified as a ‘response’ trait as it provides an indication of the vulnerability of a given organism to physical disturbance. For example, an organism that is permanently attached to a substrate is more vulnerable to disturbance than a free-living or even temporarily attached organism. (**Pa**) As degree of attachment is an expression of both structural and morphological properties of an organisms which are known to remain relatively constant over time (Volaire et al., 2020), degree of attachment can be classified as a ‘pattern’ trait. (**M**) Lastly, degree of attachment can be regarded as a morphological trait, as whether an organism exhibits no, temporary, or permanent attachment is an expression of its morphology.

**Body flexibility:** (**R**) this trait can be classified as a ‘response’ trait as it provides an indication of the vulnerability of an organism to mechanical disturbance. An organism with limited or no flexibility is more prone to lasting damage than a highly flexible organism. (**Pa**) Owing to the fairly static nature of morphological and structural features, body flexibility can be regarded as a ‘pattern’ trait (Volaire et al., 2020). (**M**) Lastly, the body flexibility of an organism is an expression of its morphology.

**Sociability:** (**R–E**) this trait can be classified as a ‘response trait’ as it provides an indication of how much of a reduction in organism numbers can be expected following physical disturbance. Benthic, sedentary organisms that tend to cluster with conspecifics are more likely to suffer substantial losses in term of population size following physical disturbance, e.g., trawling or dredging, relative to solitary organisms. Sociability can also be classified as an ‘effect’ trait, as the function/role that an organism plays within the ecosystem is magnified by a gregarious behaviour when compared to solitary organisms (Campanyà-Llovet et al., 2023). (**Pa**) Sociability can be classified as a ‘pattern’ trait as it could easily be measured under optimised, standardised conditions (e.g., in laboratory settings), but it may vary spatially along a resource gradient. For example, organisms may cluster in nutrient-rich waters due to a lack of competition and they may occur in overdispersed patterns in nutrient-poor environments due to increased competition for resources. (**Pr**) Sociability could further be argued to fall under the category of ‘process’ trait, as the tendency to cluster may vary over an organism’s life span. For example, it has been suggested that glass sponges may not compete as juveniles but, as they increase in size and require more resources, competition with neighbours results in a reduction or thinning of the density (Mitchell and Harris, 2020). (**B**) Lastly, sociability is an example of a ‘behavioural’ trait as it may vary over time as a result of both external, e.g., threats from other organisms, sound and olfactory stimuli, and internal stimuli, e.g., hunger.

**Body protection:** (**R**) this trait can be classified as a ‘response’ trait as it provides an indication of the susceptibility of an organism to changes in ocean chemistry, e.g., pH and carbonate saturation states (Costello et al., 2015), as well as providing an indication of palatability (Degen and Faulwetter, 2019) and serving as a proxy for carbon sink in species with calcium carbonate skeletons (Rossi, 2013). (**Pa**) Body protection can also be classified as a ‘pattern’ trait, as, for example, the nature of an organism’s skeleton can be measured under optimal conditions, but its integrity may vary along spatial, environmental gradients. Morphological traits are also fairly ‘static’ and tend to fall under the ‘pattern trait’ category (Volaire et al., 2020). (**M**) Lastly, the body protection of an organism is an expression of its morphology.

**Age at sexual maturity:** (**R**) this trait can be classified as a ‘response’ trait as it provides an indication of how likely an organism is to recover following mechanical disturbance. Organisms that mature early are more likely to recover following disturbance relative to organisms that mature later in life (*r* versus *K* strategists). (**Pa**) Age at sexual maturity can also be classified as a ‘pattern’ trait, as such traits are predominantly morphological, structural, or physiological in nature, shaped by evolutionary processes and accounting for a substantial share of the variation in resource acquisition and/or resource conservation. (**L**) Lastly, age at sexual maturity is an example of a ‘life history’ trait, as it pertains to a life strategy resulting from evolutionary convergences which, in turn, result from universal energetic allocation trade-offs (Greenslade, 1983; Southwood, 1988; Kindsvater et al., 2016).

**Body form:** (**R–E**) body form suggests vulnerability to mechanical disturbances (‘response’) and relates to potential roles that the organisms can play within an ecosystem (i.e., habitat-forming, ‘effect’). Organisms characterised by an upright, globular, elongated, and laterally compressed body form are more exposed to mechanical disturbance than dorsoventrally compressed organisms. However, the former are also more likely to act as a potential habitat or refuge for other organisms, e.g., corals (Darling et al., 2012; Campanyà-Llovet et al., 2023). (**Pa**) Owing to the fairly static nature of morphological features, body form can be regarded as a ‘pattern’ trait (Volaire et al., 2020). (**M**) Lastly, the body form of an organism is an expression of its morphology.

## **References**

Bellwood, D. R., Streit, R. P., Brandl, S. J. and Tebbett, S. B. (2019) ‘The meaning of the term “function” in ecology: A coral reef perspective.’ *Functional Ecology*, 33(6) pp. 948–961.

Campanyà-Llovet, N., Bates, A. E., Cuvelier, D., Giacomello, E., Catarino, D., Gooday, A. J., Berning, B., Figuerola, B., Malaquias, M. A. E., Moura, C. J., Xavier, J. R., Sutton, T. T., Fauconnet, L., Ramalho, S. P., Neves, B. de M., Machado, G. M., Horton, T., Gebruk, A. V., Minin, K., Bried, J., Molodtsova, T., Silva, M. A., Dilman, A., Kremenetskaia, A., Costa, E. F. S., Clarke, J., Martins, H. R., Pham, C. K., Carreiro-Silva, M. and Colaço, A. (2023) ‘FUN Azores: a FUNctional trait database for the meio-, macro-, and megafauna from the Azores Marine Park (Mid-Atlantic Ridge).’ *Frontiers in Ecology and Evolution*, 11.

Costello, M. J., Claus, S., Dekeyzer, S., Vandepitte, L., Tuama, É. Ó., Lear, D. and Tyler-Walters, H. (2015) ‘Biological and ecological traits of marine species.’ *PeerJ*. PeerJ Inc., 3, August, p. e1201.

Darling, E. S., Alvarez-Filip, L., Oliver, T. A., McClanahan, T. R. and Côté, I. M. (2012) ‘Evaluating life-history strategies of reef corals from species traits.’ *Ecology Letters*, 15(12) pp. 1378–1386.

Degen, R. and Faulwetter, S. (2019) ‘The Arctic Traits Database – a repository of Arctic benthic invertebrate traits.’ *Earth System Science Data*. Copernicus GmbH, 11(1) pp. 301–322.

Greenslade, P. J. M. (1983) ‘Adversity Selection and the Habitat Templet.’ *The American Naturalist*. The University of Chicago Press, 122(3) pp. 352–365.

Kindsvater, H. K., Mangel, M., Reynolds, J. D. and Dulvy, N. K. (2016) ‘Ten principles from evolutionary ecology essential for effective marine conservation.’ *Ecology and Evolution*, 6(7) pp. 2125–2138.

Mitchell, E. G. and Harris, S. (2020) ‘Mortality, Population and Community Dynamics of the Glass Sponge Dominated Community “The Forest of the Weird” From the Ridge Seamount, Johnston Atoll, Pacific Ocean.’ *Frontiers in Marine Science*. Frontiers, 7, October.

Rossi, S. (2013) ‘The destruction of the “animal forests” in the oceans: Towards an over-simplification of the benthic ecosystems.’ *Ocean & Coastal Management*, 84, November, pp. 77–85.

Southwood, T. R. E. (1988) ‘Tactics, Strategies and Templets.’ *Oikos*. [Nordic Society Oikos, Wiley], 52(1) pp. 3–18.

Volaire, F., Gleason, S. M. and Delzon, S. (2020) ‘What do you mean “functional” in ecology? Patterns versus processes.’ *Ecology and Evolution*, 10(21) pp. 11875–11885.

| **Trait**  **Table A1.** List of 18 commonly used traits in benthic research with associated synonyms and modalities. Red text indicates usage in the BIOTIC database (*n* = 407). | **Synonyms** | **Modalities** |
| --- | --- | --- |
| **Longevity** | Life span  Lifespan  Life-span  Adult longevity  Adult life span  Life duration  Maximum longevity  Maximum lifespan  Maximum life span  Max age | Age classes (numerical or ordinal) |
|  |  |  |
| **Body size** | Size  Body length  Maximum body size  Maximum body length  Maximum length  Maximum size  Maximal size  Mean (adult) size  Mean body size  Average size  Size (Height)  Size (Maximum width)  Size range  Estimated maximum body size  Body-size  Body-mass  Body mass  Normal adult size  Adult size  Adult body mass/size  Maximum adult size  Maximum adult body size  Maximum weight  Max. body length  Individual/colony size  Individual size  Maximum colony size  Potential size  Maximum potential size  Organism body size  Maximum size of organism  Normal size | Length/weight classes (numerical or ordinal), XS–XL |
|  |  |  |
| **Mobility** | Adult mobility  Adult motility  Motility  Motility level  Relative adult mobility  Adult mobility (relative)  Movement method  Moving mode  Adult movement  Adult movement method  Adult moving  Movement  Movement type  Movement ability  Type of movement  Degree of motility  Daily adult movement capacity  Living habit  Mobility within sediment  Mobility in sediment  Movement in the sediment  Primary locomotion  Locomotion  Adult locomotion  Post-settlement mobility  Daily movement capacity  Mobility of adult | (Temporarily/temporary/permanent) attach(ed/ment) (temporary, permanent/nearly)/sessile, attached-sessile, tubicolous, crawl(er), (free) crawl(er/ing), swim(mer/ming), flyer, (free) burrow(er/ing), creep(er), climb(er), jumper, tube dwell(er/ing), drifter, crevice, walk(er), burrower & swimmer, walker & swimmer, gliding, crawler-swimmer, planktonic, byssus—  Highly mobile, mobile/motile, discretely/moderately/semi mobile/semi-motile, sedentary, sessile/immobile (ordinal classes), passive, passively/facultatively/intermittently/habitually mobile, vertical migrator, mobile resident, horizontal migrator, nomadic, live in fixed tube, limited movement, slow movement (in sediment matrix), free movement in burrow system, ordinal classes (none, low, medium, high), limited free movement, freely motile in/on sediment, semi-pelagic, non-motile/semi-motile, vagile |
|  |  |  |
| **Feeding mode** | Feeding  Feeding habit  Feeding habits  Feeding method  Feeding mechanism  Feeding strategy  Feeding-strategy  Feeding type  Feeding-type  Feeding guild  Feeding behaviour  Feeding location  Feeding ecology  Feeding position  Characteristic feeding method  Resource capture method  Adult feeding habit  Adult feeding habitat  Adult feeding  Trophic group  Trophic groups  Trophic mode  Trophic position  Primary trophic mode  Feeding group  Diet  Food method and habits  Foraging strategy  Dominant feeding method | Deposit feed(ing/er), deposit-suspension feed(ing/er), (active/passive/microphage) suspension feed(ing/er)/suspensivore, filter feed(ing/er), carnivor(e/y/ous), omnivore(e/y/ous), opportunist/scavenger, herbivore, herbivorous, grazer, (microphage) surface deposit feeder, (microphage) sub-surface deposit feeder, predator, parasit(e/ic), detrivore, detritus feed(-ing/-er)(/sandlicker), deposit-feeder, filter-feeder, bacterivore, browser, macrophage, photosynthetic, photoautotroph, corallivores, micro/macro-invertivores, planktivores, piscivores, microbivore, microbivore and carnivore, symbiont contribution, planktotroph, chemoautotroph, interface feeder, grazer (grains/particles), grazer (fronds/blades), grazer (surface/substratum), surface-deposit feeder, subsurface-deposit feeder, carnivore/omnivore, symbiont, filter/suspension feeder, herbivore/opportunist/scavenger, deposit/sediment/periphyton feeder, mixtes, parasite/endosymbiont, wood-boring, selective (bacterial) feeders, non-selective deposit feeders, epistrate or epigrowth feeders, predators/omnivores |
|  |  |  |
| **Reproductive mode** | Reproductive technique  Reproductive type  Reproduction  Reproduction/development mode  Reproductive strategy  Reproductive strategies  Reproductive guild  Reproduction strategy  Reproduction mode  Reproduction type  Reproductive method  Reprod technique  Mode of reproduction  Egg development location  Egg development  Development strategy  Developmental technique  Developmental mechanism  Developmental mode | Asexual (budding), sexual (broadcast spawner/pelagic), sexual (ovigerous/egg lay/brood-planktonic larvae, benthic/demersal eggs), sexual (egg lay/brood-mini adults/direct development/viviparous), sexual dioecious, sexual hermaphrodite, colonial, fission, parthenogenesis, permanent/protandrous/protogynous hermaphrodite, gonochoristic, vegetative, self-fertilization, alternation of generations, isogamous, anisogamous, oogamous, pelagic, short-pelagic, benthic, ovi/ovoviviparous, spawn, attached eggs, brood, planktotrophic larvae, lecithotrophic larvae, brood to larvae, brood to juvenile, free spawning, eggs laid in capsules or masses |
|  |  |  |
| **Sexual differentiation** | Reproduction  Reproductive type  Reproductive technique  Sex strategy  Sexual expressions | Asexual (reproduction), gonochoric/gonochoristic, (sequential/synchronous, simultaneous) hermaphrodite, alternate, dioecious, protandry (male first), protogyny (female first) |
|  |  |  |
| **Reproductive frequency** | Reproduction frequency  No of reprod per year  Lifetime no. reproductive  Opportunities  Reprod. Frequency  Reproductive strategy of the  individuals | Seasonal, (semi-) continuous, iteroparous (polytelic), semelparous (monotelic), annual (2 or more reproductive events), annual (1 reproductive event/once), annual episodic, annual protracted, biennial, numerical, biannual episodic, biannual protracted, < biannual, number of reproductive opportunities, twice every year, once per year (extended period), once per year (distinctive period), once per 2 years (extended period) |
|  |  |  |
| **Fragility** | Structural robustness  Structural fragility  Flexibility/Fragility | Robust, strong, intermediate, (very) fragile/highly breakable/limited flexibility, soft and flexible, hard shell (protection), rigid exoskeleton, burrow, vermiform, regeneration; strong, flexible; no protection; fragile shell/structure; not known |
|  |  |  |
| **Developmental mechanism** | Larval development  Larval-development  Larval development location  Larva development location  Larval mode of development  Development mode  Development mechanism  Development stage  Mechanism of development  Early development  Early development mode  Larvae type  Larvae  Larval type  Type of larvae  Larval development mode  Larval development strategy  Larval environmental development  Larva  Propagule dispersal  Developmental type  Larval diet  Larval mode  Reproductive mode (larval  development) | Pelagic, benthic, direct (without/no larval stage)—Planktotrophic, lecithotrophic, benthic/benthonic/direct development (no larval stage), fragmentation/fission, oviparous, ovoviviparous, viviparous (parental care), viviparous (no care), brooding, spores (sexual/asexual), epitokia, brood to larva, brood to juvenile, asexual budding, veliger, zoea larvae, trochophore larvae, other types of larvae |
|  |  |  |
| **Living habit** | Adult habitat  Habitat  Adult life habitat  Life habitat  Life habit  Living-position  Living position  Living habitat  Living mode  Living strategy/mode  Lifestyle  Local habitat  Habitat structure  Habitat type  Adult life habit  Habit  Habitat creation ability  Habitat complexity  Habitat (living position)  Environmental position  Tiering types | (Permanent/semi-permanent) tube(-dwelling/dweller/builder), (permanent/temporary) burrow(-dwelling/dweller), free-living, crevice(-dwelling)/hole/understone/sheltered refuge, epi/endo zoic/phytic, (permanent/temporarily) attached (to bed/substratum), (surface) crawler, swimmer, parasite/commensal, attached, bed forming, ectoparasitic, encrusting, erect, reef building, tubicolous, surface crawler/swimmer, pelagic, epi/hyperbenthic, endo/infauna, fixed, tube/borer, maintained burrow, unmaintained burrow, epifaunal attached, epifaunal free living, semi-shallow infaunal, mid-deep infaunal, tube permanent attachment, tube semi-permanent attachment, errant |
|  |  |  |
| **Bioturbation mode** | Bioturbation  Bioturbatory mode  Bioturbatory activity  Bioturbator  Bioturbation type  Bioturbation activity  Sediment transport  Sediment mixing  Sediment moving  Sediment reworking  Sediment reworking mode  Sediment reworking types  Surface sediment reworking  Reworking types  Reworking type  Reworking  Reworking mode  Mode of bioturbation  Method of bioturbation  Biomixing  Biotransport  Microhabitat use  Bioturbation and bioirrigation  Bioturbation functional type | Diffusive mixing, surface deposition, upward conveyor, downward (reverse) conveyor, up/down conveyor, non-bioturbator, surficial/superficial modifiers, biodiffus(e/o)rs, regenerators, epifauna, conveyer belt transport, reverse conveyer belt transport, gallery diffuser, bio(-)irrigator, surface mixing, surface deposition, deep(er) mixing, transport, tube-dweller, vertical transporter, diffusion, advection, surface-to-deep, deep-to-surface, surficial reworking; ordinal classes (high, medium, moderate, low-none) |
|  |  |  |
| **Environmental position** | Sediment position  Adult preferred substrate position  Preferred substrate position  Position  Living location/environmental  position  Living position  Living-position  Living location/position  Life zone  Depth penetration in the sediment  Depth  Position in sediment  Position on the substrata/um  Position on the seabed  Habitat  Habitat position  Habitat (living position)  Sediment dwelling depth  Living position in habitat  Benthic position in sediment  Environmental positioning  Environment position  Position in the sediment  Position in sediments  Position of organisms in the  sediment  Positioning in the sediment  Depth in the sediment/burrowing  capacity  Benthic position  Adult environmental position  Location within sediment  Location in or on sediment  Burrowing depth  Substratum depth distribution  Burrow type  Sediment localization  Substrate position  Seabed position  Living position on the substratum  Living position in sediment  Sediment depth | Depth classes, surface (dwelling/burrowing), infauna, infaunal (top/bottom, shallow/mid-depth/deep), attached, within plants (unattached), benthic, bentho(-)pelagic, pelagic, epibenthic/epibenthic and interface/epibiont(e), hyperbenthic, endofauna/endobenthic, crevices/stones/shells, throughout sediment column, middle, deep/deep burrowing, emergent, epifaunal, epifloral, interstitial, demersal, epilithic, epiphytic, epizoic, lithotomous, tube dwelling, burrow dwelling, free living, attached, surface (epibenthic), shallow infauna (<5 cm), deep infauna, fixed, protruding surface, attached to hard substrate or other structures, oxygenated zone, below oxygenated zone |
|  |  |  |
| **Degree of attachment** | Degree of attachment to substrate  Strength of attachment  Attachment  Adult attachment  Attached  Degree of dependency | None, temporary, permanent |
|  |  |  |
| **Body flexibility** | Flexibility  Degree of flexibility | Degree classes, ordinal classes (high, low, none/not flexible, limited flexibility, highly flexible) |
|  |  |  |
| **Sociability** | Gregariousness  Degree of contagion  Colony formation  Coloniality  Solitary-colonial  Intra-specific sociability  Modularity  Aggregation  Grouping habits | Solitary, patchy, gregarious, facultative gregarious, highly aggregated/colonial, pairing, living in small groups (<20 individuals), schooling species (>20 individuals), ordinal classes (never, sometimes, always), aggregation, aggregation of a few individuals |
|  |  |  |
| **Body protection** | Skeleton  Skeleton presence  Skeleton material  Skeletal composition  Protection  Rigidity  Morphology  Physical defences/calcification  Body texture  Body hardness  Hardness  Protective structure  External protection | (Unprotected) soft(-bodied/tissue)/fragile, soft-protected, tunic, (thin/hard) exoskeleton, brittle, crustose, cushion, stalked, calcareous (aragonite, calcite), chitinous, silicious, cuticle, exoskeleton, endoskeleton, plant cell wall, (thin/soft/hard) shell, durable/flexible, no hard skeleton, hard skeleton, irregular, round, vermiform, rigid/rigid tubes, non-calcareous spicules, calcareous spicules and sclerites, external carbonate, carbonate with discontinuities, continuous carbonate, no protection, tube, shell(-ed), amorphous calcium carbonate, calcified, calcium carbonate, bone mineral, cartilage, none, low protection, tubiculous |
|  |  |  |
| **Age at sexual maturity** | Age at maturity  Time to maturity  Age at reproductive maturity  Age at which sexual maturity is reached  Age to first reproduction/generation time  Age at first reproduction  Age of maturity  Sexual maturity  Time to reproductive maturity  Mature years  Maturity  1^st^ age of reproduction | Age classes |
|  |  |  |
| **Body form** | Skeleton  Rigidity  Morphology  Morphological form  Growth form  Body texture  Body design  Body shape  Body type  Bodyform  Adult body form  Adult body shape  Adult shape | Soft(-bodied/protected)/fragile, tunic, exoskeleton, crustose (hard/soft), cushion, stalked, erect/upright, conical, elongated, streamlined, spherical (round body)/globulose/ball-shaped/globose/globular, mound, low profile (thin/long and thin/threadlike), flat, flattened (body) dorsally/dorsoventrally/dorso-ventral compressed, flattened (body) laterally/laterally compressed, hard, hard exoskeleton, hard shell, irregular, vermiform (unsegmented/segmented/annulated), rigid/rigid tubes, short cylindrical/cylindric body, boring, filaments, stolonial, encrusting, encrusting leaf-life with blades, foliose erect thallus, coarsely branched, articulated, cup-like, massive encrusting, massive hemispheric, massive-erect, tree-like, boring, flaccid, turf, shrub, arborescent/arbuscular, forest, algal gravel, accretion, mat, faunal beds, radial, stellate, whiplike, straplike/ribbonlike, filiform/filamentous, digitate, lanceolate, penicillate, pinnate, capitate/clubbed, clathrate, reticulate, funnel shaped, dendroid, flabellate, tubicolous, medusiform/medusoid, cylindrical, bullate/saccate, bivalved, turbinate, conical, vermiworm (segmented), vermiworm (unsegmented), colonial, others, stout, slender, long/thin |
